# Carbon source–driven metabolic and regulatory remodeling defines phenomic states in *Lipomyces starkeyi*

**DOI:** 10.1038/s41598-026-53531-2

**Published:** 2026-08-12

**Authors:** Lummy M. O. Monteiro, Xiaolu Li, Kyle R. Pomraning, Jasmin Alvarez, Song Feng, Teresa Lemmon, Marie Swita, Heather Olson, Josie G. Eder, Tong Zhang, Sneha Couvillion, Jason E. McDermott, Jeffrey J. Czajka

**Affiliations:** 1 Earth and Biological Sciences Directorate, Pacific Northwest National Laboratory, Richland, WA USA; 2 Energy and Environment Directorate, Pacific Northwest National Laboratory, Richland, WA USA; 3 Department of Molecular Microbiology and Immunology, Oregon Health and Science University, Portland, OR USA

**Keywords:** Predictive phenomics, Oleaginous yeast, Multi-omic data, Biotechnology, Biochemistry, Biotechnology, Computational biology and bioinformatics, Systems biology

## Abstract

*Lipomyces* is a genus of oleaginous yeasts with potential for contributing to reliable biomanufacturing supply chains. However, progress in advanced strain designs and engineering efforts are still constrained by a lack of understanding of the underlying molecular drivers of *Lipomyces* phenotypes. To address this gap, we collected a suite of multi-omic data to dissect how carbon source availability reshapes the metabolic network, lipid allocation, and regulatory architecture of *Lipomyces starkeyi*. We observed that glucose promotes biosynthetic and proliferative processes supported by abundant energy and carbon intermediates, xylose enhances redox-balancing mechanisms centered on the pentose phosphate pathway, and glycerol activates respiratory metabolism, β-oxidation, and the glyoxylate cycle. Lipid species distributions remained consistent in both nitrogen replete and depleted conditions across the carbon sources, indicating robust production mechanisms. Regulatory protein identification and network analysis revealed glycerol-driven respiratory growth favors regulatory programs integrating stress tolerance, redox balance, and lipid-associated metabolism, whereas xylose growth activates compensatory transcriptional responses aimed at maintaining mitochondrial function. Nitrogen limitation modulates the strength of these responses but does not fundamentally alter their direction, reinforcing carbon source as the dominant driver of regulatory architecture. Taken together, this data enhances the understanding of *Lipomyces* molecular rearrangements and provides a foundation for further development of predictive phenotypic tools in this genus.

**Supplementary Information:**

The online version contains supplementary material available at 10.1038/s41598-026-53531-2.

## Introduction

Oleaginous microorganisms are species that can accumulate more than 20% of their dry cell weight (DCW) as lipids under specific culture conditions. These microbes are attractive platforms for production of chemicals and fuels due to their naturally high acetyl-CoA flux and lipid content. *Lipomyces* are a particularly promising oleaginous genus of yeast that are relatively easy to cultivate and are among the highest natural accumulators of lipids, with certain species obtaining over 60% lipid content^1^. Despite this potential, *Lipomyces* has seen limited development as an industrial chassis due to a lack of robust engineering tools. However, this has been changing as genetic engineering capabilities for *Lipomyces* species have advanced considerably over the last decade^2–5^. Proof-of-concept work has demonstrated re-wiring of *L. starkeyi*^6^ and *L. tetrasporus* metabolism to produce the platform chemical malic acid and *L. starkeyi* to produce a sesquiterpenoid^7^. At the same time, recent genomic sequencing of 25 yeasts in the *Lipomyces* clade, along with the corresponding development of a *L. starkeyi* genome-scale model (GSM)^8^, are now facilitating computational tools for designing strains constructs. Together, these developments have positioned *Lipomyces* as a potential biomanufacturing production chassis.

However, progress in advanced strain designs and engineering efforts are still constrained by a lack of the underlying molecular drivers of *Lipomyces* phenotypes, with a limited system-level understanding of how carbon source availability reshapes metabolic flux distribution, lipid allocation, and regulatory architecture. This information is essential for understanding the lipogenic process, stress response, oleochemical production, and other key pathways (recently reviewed^9^. Compared to model oleaginous yeasts such as *Yarrowia lipolytica* and *Rhodotorula toruloides* (previously *Rhodosporidium toruloides*), *Lipomyces* remains underrepresented in omics research^9^. While these model organisms have been extensively characterized through multi-omic and systems biology approaches, enabling detailed understanding of their metabolic and regulatory networks, comparable systems-level datasets for *L. starkeyi* remain limited. This gap constrains the ability to interpret its phenotypic behavior and to rationally engineer this organism for biotechnological applications. Early proteomic work in *Lipomyces* identified 289 proteins over the course of growth^10^, while more recent efforts have generated transcriptomic, metabolomic, and proteomic data during hydrolysate fermentations^6^. In contrast, *Y. lipolytica* has been extensively profiled using multi-omic techniques, yielding actionable insights—for example, identifying ketogenic amino acid metabolism as a key contributor during docosahexaenoic acid production and enabling feeding strategies that increased titers by 40%^11^. Another study paired multi-omic data with a Bayesian modeling approach to identify key enzymes controlling itaconic acid^12^. Post-translation modifications (PTMs) research has further shown that phosphorylation of storage lipid enzymes can regulate the nitrogen limitation response and influence lipid production^13^. Similar PTM and omic-focused investigations in *R. toruloides* have characterized redox and phosphorylation changes during nitrogen limitation^14^. Collectively, these examples illustrate how multi-omic derived knowledge can accelerate metabolic engineering efforts and highlight the opportunity for comparable analyses in *Lipomyces*.

Here, we focused on the non-mucus producing mutant *L. starkeyi* NRRL Y-11558^3^. We investigate its molecular responses and control points during growth on media compositions relevant to waste feedstocks, such as hydrolysates and glycerol. We dissect how carbon source availability rewires the metabolic network, lipid allocation, and regulatory network architecture. By integrating lipidomics, proteomics, metabolomics, and post-translational modification datasets across glucose, xylose, and glycerol growth conditions – in both nitrogen sufficient (replete) and limited (depleted) conditions – we reveal a coordinated substrate-specific metabolic and regulatory landscape. Our results establish carbon source as the primary driver of proteomics and metabolic state, define a continuum from fermentative to oxidative metabolism, and identify regulatory hubs that interface central carbon metabolism with lipid pathways and stress responsive networks. Together, these findings provide a systems-level frameworks for understanding and engineering lipid metabolism in *Lipomyces*.

## Results

### Phenotypic/growth data

**Fig. 1:**
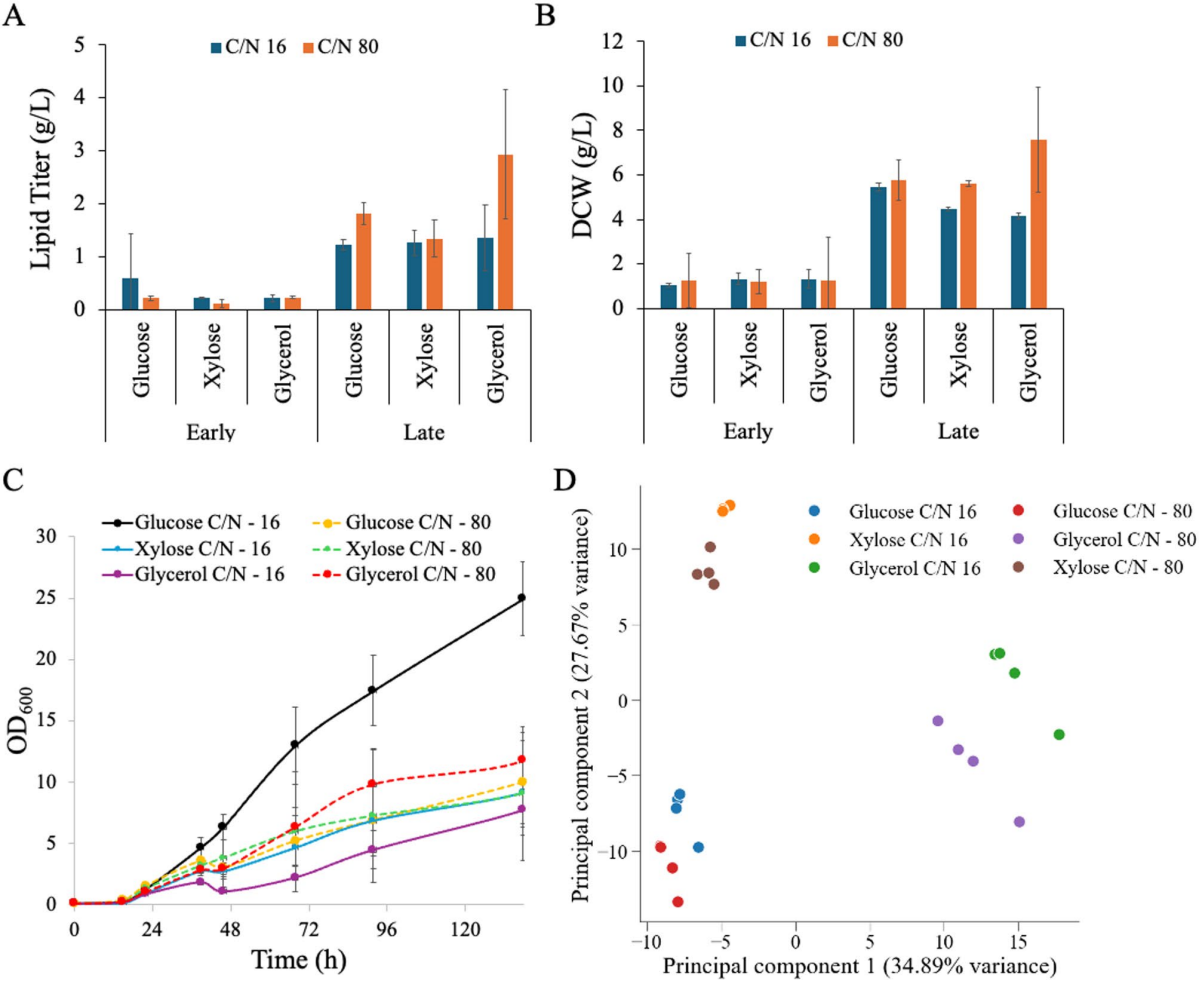
Phenotypic outcomes of *L. starkeyi* on various carbon sources in nitrogen replete (C/N 16) and nitrogen deplete (C/N 80) conditions. (**a**) Lipid Titer. (**b**) Dry cell weight. (**c**) Growth curves. (**d**) Late stage proteomic PCA plot.

We utilized common hydrolysate carbon sources (glucose and xylose) and the industrial waste carbon source (glycerol) at a C/N ratio of 16 and 80, representing nitrogen rich (replete) and limited (deplete) medias. With 40 g/L of carbon, *L. starkeyi* obtained OD_600_ values between 10 and 20 in our growth timeframes (Fig. 1). With this information in mind, we grew our strains in these medias and evaluated the lipid and DCW titers, shown for the three main carbon sources (Fig. 1). During the early exponential growth phase, the cells obtained lipid yields of 14 ± 6%, 17 ± 4%, and 16 ± 1% from glucose, xylose, and glycerol, respectively, in the nitrogen replete conditions (Supplementary Table S1). The lipid content (as measured by fatty acid methyl ester (FAME)) was increased in our nitrogen limited conditions at later timepoints, where we obtained lipid yields of 32 ± 2%, 24 ± 6%, and 38 ± 4% from glucose, xylose, and glycerol, respectively (Supplementary Table S1). Our multi-omic data samples were then collected from these conditions to evaluate the molecular signatures responsible for growth and to dissect processes that may regulate lipid accumulation during nitrogen deplete conditions.

### Carbon source shapes proteomic remodeling across a metabolic continuum from fermentation to oxidation

As carbon source dictates the balance between fermentative and oxidative metabolism, it is expected to define dominant proteomic states, with nitrogen availability acting as a secondary modifier. We therefore collected multi-omic data from all conditions at OD_600_ values of approximately one and between four to five. Our data indicated relatively limited remodeling of cellular processes between the two doublings at the protein abundance level. Our main results discussed here are compiled using the early exponential phase timepoints, where metabolic differences across carbon sources are most clearly resolved. Although additional timepoints were collected throughout growth and are provided in the supplementary materials, the major carbon source–dependent proteomic and lipidomic trends remained consistent over time, with only quantitative differences observed between growth stages. Comparing the global proteomic profiles across glucose, xylose, and glycerol conditions under both basal and nitrogen-limited media revealed a clear pattern: samples tended to cluster primarily according to the carbon source rather than nitrogen availability (Fig. 1 and Supplementary Fig. S1). This trend was evident in the principal component analysis (PCA), where nitrogen-replete and nitrogen-limited samples clustered together within each carbon source, suggesting similar proteomic responses, while samples grown on different carbon sources remained well separated (Fig. 1 and Supplementary Fig. S1). Differential protein abundance was assessed using adjusted p-values and fold-change thresholds as described in the Supplementary Methods. Proteins were considered significantly regulated using an adjusted p-value < 0.05 combined with a stringent fold-change cutoff (|log₂FC| ≥ 1), prioritizing robust and high-confidence abundance changes across conditions. Across conditions, a total of 2588 proteins were consistently quantified, of which 12–135 proteins were differentially abundant depending on the comparison, with the number of regulated proteins varying by carbon source and nutrient regime (Supplementary Figs. S1–S2). Consistent with the PCA, differential protein abundance analyses revealed that carbon source–specific proteomic signatures were largely preserved regardless of nitrogen status. For each carbon source, a substantial subset of proteins exhibited similar directional changes under both nitrogen-replete and nitrogen-limited conditions, forming reproducible carbon source–associated fingerprints. In contrast, comparisons between nitrogen-replete and nitrogen-limited states within the same carbon source revealed fewer shared changes, indicating that nitrogen availability modulates the magnitude of proteomic responses without redefining the underlying carbon-driven metabolic programs (Supplementary Fig. S2).

Together, these patterns indicate that metabolic adaptation in *L. starkeyi* is primarily structured by carbon source, with nitrogen availability acting as a secondary modifier. This behavior is consistent with the expectation that the core enzymatic machinery required for carbon assimilation and central metabolic routing remains conserved under nitrogen limitation, while growth rate and biosynthetic capacity are adjusted accordingly.

**Fig. 2:**
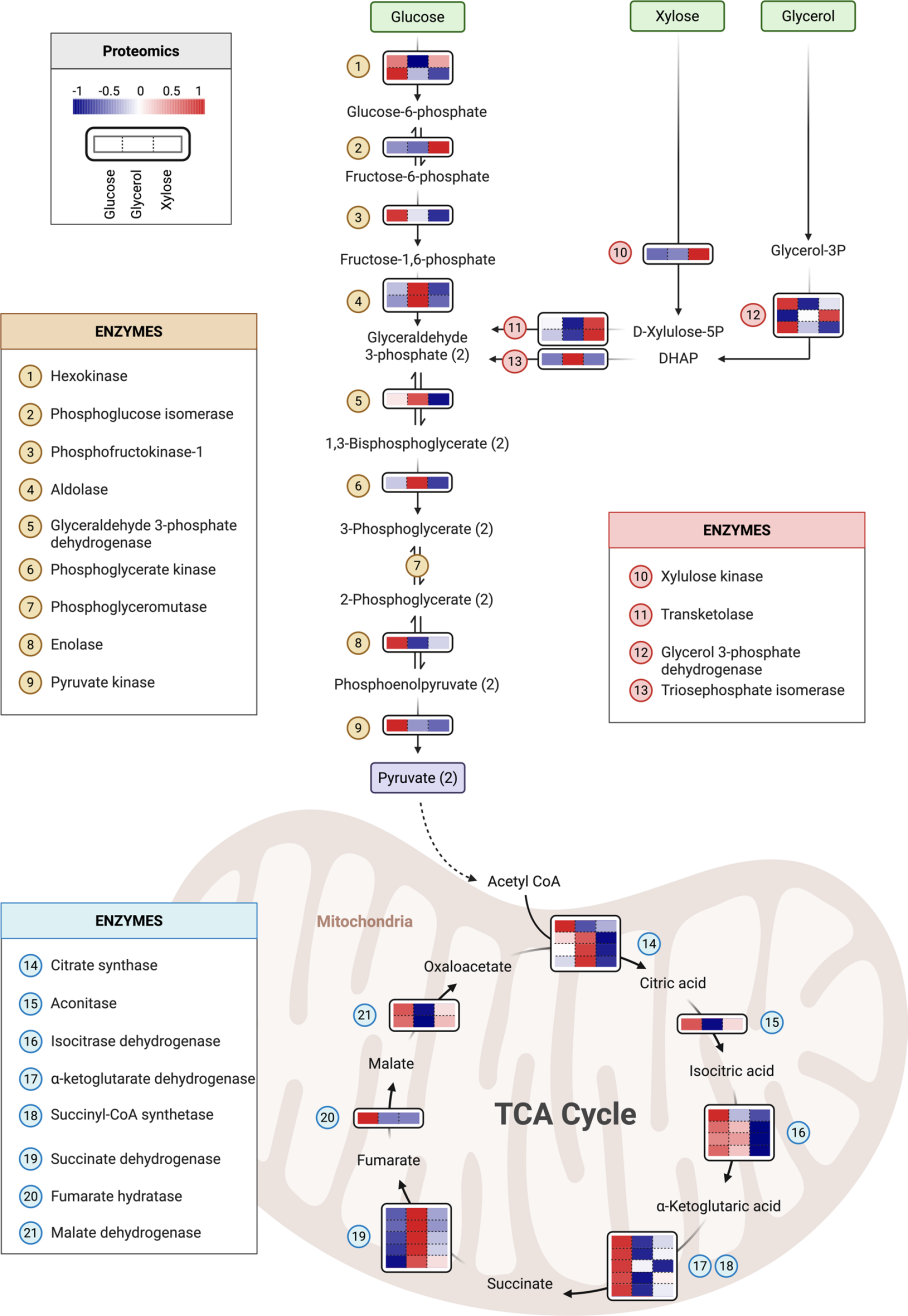
Proteomic remodeling of central carbon metabolism across distinct carbon sources. Schematic representation of glycolysis, the pentose phosphate pathway (PPP), and the tricarboxylic acid (TCA) cycle highlighting relative protein abundance changes under glucose, xylose, and glycerol growth conditions. Heatmaps associated with individual enzymatic steps depict normalized proteomic abundance patterns across conditions. For each protein, values represent the mean of all biological replicates for each condition. Color scales represent row-wise z-score–scaled group means, illustrating carbon source–dependent shifts from fermentative glycolysis toward oxidative TCA metabolism. Enzymes are annotated by pathway and reaction step, with color gradients indicating differential protein abundance across carbon sources, as derived from quantitative proteomics. Created in BioRender. Oliveira Monteiro, L. M. (2026) https://BioRender.com/fmwgmu6.

To better interpret the biological basis of the carbon source–driven separation, we investigated the proteomic dataset to understand how key metabolic nodes were reprogrammed under glucose, xylose, and glycerol growth conditions (Fig. 2). The combined profiles revealed a coordinated yet substrate-specific metabolic reconfiguration, reflecting *L. starkeyi* capacity to modulate carbon flux distribution, redox balance, and biosynthetic priorities in response to carbon availability. Glucose-supported growth exhibited a trend toward higher abundance of glycolytic enzymes and intermediates compared with cells grown on xylose or glycerol, consistent with an anabolic, fermentative-like metabolism. Xylose induced a mixed state enriched in pentose phosphate pathway (PPP) and redox-related components, suggesting enhanced NADPH generation and adaptive control of redox homeostasis consistent with transcriptome remodeling during transition from glucose to xylose consumption^15^. In contrast, glycerol-grown cells displayed elevated levels of TCA cycle enzymes and metabolites, indicative of stronger oxidative metabolism and enhanced anaplerotic activity. Together, these patterns delineate a metabolic continuum linking carbon source utilization to fundamental shifts in energy metabolism and biosynthetic investment. These trends were consistent under both base and nitrogen-limited conditions (Supplementary Fig. S3), reinforcing our observation that carbon source, rather than nitrogen status, is the primary determinant of metabolic remodeling in this system and conditions. To confirm that the observed patters reflected biologically meaningful responses rather than global statistical separation of samples, we examined the expression of individual proteins within each carbon source. These pathway-level trends are explored in detail in the following subsections, where we highlight how each carbon source imprints a characteristic metabolic signature that supports its known biological function.

### Glucose induces an anabolic and growth-promoting proteomic state

Under glucose-supported growth, the proteome displayed a pronounced fermentative and anabolic metabolic profile, centered on glycolysis-derived carbon flux and associated biosynthetic pathways. Key enzymes linking central carbon metabolism to amino acid biosynthesis were significantly upregulated (adjusted p-value < 0.05 and (|log₂FC| ≥ 1)), particularly within the serine–cysteine–methionine axis. These included D-3-phosphoglycerate dehydrogenase, serine O-acetyltransferase, methionine synthase II, cysteine desulfurase (NFS1), ATP sulfurylase, and adenosine 5′-phosphosulfate kinase, highlighting the channeling of glycolytic intermediates toward sulfur assimilation, cysteine and methionine synthesis, and downstream processes such as Fe–S cluster assembly and methyl-group metabolism. This metabolic state was accompanied by the induction of phosphoglucomutase, pfkB-like carbohydrate kinases, and aldo/keto reductases suggests extensive carbohydrate interconversion and NADPH-dependent redox balancing, supporting high biosynthetic demand under fermentative growth. These proteomic signatures are consistent with an anabolic, glucose-driven metabolic state optimized for biomass accumulation and precursor supply, providing the metabolic baseline against which alternative carbon sources are contrasted (Supplementary Table S2).

### Xylose metabolism activates redox balancing and pentose phosphate flux

Global proteomic profiling under xylose cultivation confirmed the metabolic shift toward pentose phosphate pathway (PPP) activation and redox control. Enzymes such as D-ribulose-5-phosphate 3-epimerase, ribokinase, and ribulose/sugar kinases were strongly induced, suggesting active assimilation of xylose into the PPP to generate NADPH and biosynthetic precursors. Upregulation of oxidoreductases including sorbitol dehydrogenase, aldo/keto reductases, alcohol dehydrogenase (class V), aldehyde dehydrogenase, and NADP⁺-dependent malic enzyme, suggests reinforcement of NADPH/NAD⁺ homeostasis required for the conversion of xylose to xylitol to xylulose. Concurrent activation of aspartate aminotransferase, fumarate reductase, and malic enzyme integrates pentose-derived carbons into central metabolism, maintaining anaplerotic flux to the TCA cycle. Enhanced abundance of peroxisomal β-oxidation proteins, and monooxygenases indicates redox stress management and metabolite export during xylose catabolism. Meanwhile, induction of gluconeogenic and translational enzymes such as fructose-1,6-bisphosphatase, EF1β/δ, ribosomal proteins reflect sustained biosynthetic activity. In conclusion the proteomic profile represents a redox-adaptive metabolic state, balancing NADPH generation with carbon flux redistribution to maintain cellular homeostasis during pentose utilization (Supplementary Table S2)^16^.

### Glycerol promotes oxidative metabolism and peroxisomal remodeling

In the presence of glycerol, the proteome shifted toward oxidative metabolism, lipid β-oxidation, and peroxisomal activity, all characteristic of growth on non-fermentable carbon sources. The strong induction of acyl-CoA dehydrogenases, 2-enoyl-CoA hydratase, and carnitine O-acyltransferase (CPT2/YAT1) suggests activation of fatty acid degradation and acetyl-CoA generation through peroxisomal and mitochondrial β-oxidation. Although β-oxidation pathways were upregulated under glycerol conditions, this did not result in a reduction in overall lipid titers. This suggests the presence of active lipid turnover, where fatty acid degradation and synthesis occur simultaneously. In this context, β-oxidation-derived acetyl-CoA may be redirected toward central metabolism or recycled into lipid biosynthesis, particularly under nitrogen-limited conditions that favor lipid accumulation. Upregulation of isocitrate lyase and malate synthase confirms operation of the glyoxylate shunt, which enables carbon conservation and gluconeogenic precursor synthesis. Detection of triosephosphate isomerase and dihydroxyacetone kinase supports glycerol assimilation via dihydroxyacetone phosphate. Concurrent enrichment of catalase, flavin-containing monooxygenases, and aldo/keto reductases reflects the need for reactive oxygen species detoxification and redox balance under respiratory conditions. Induction of mitochondrial carrier proteins, aspartate aminotransferase (AAT1/GOT2), and methylmalonate semialdehyde dehydrogenase indicates integration of amino acid and lipid catabolism with central metabolism. These responses define a respiratory-oxidative proteomic state optimized for energy-efficient carbon utilization and cellular maintenance in the absence of fermentable sugars (Supplementary Table S2). This profile is consistent with oxygen-dependent respiratory metabolism under the aerobic cultivation conditions used in this study. Although oxygen levels were not explicitly controlled or varied, the observed proteomic and metabolic patterns strongly support a shift toward respiratory metabolism during growth on glycerol.

### Carbon source–driven metabolic continuum

The comparative analysis across carbon sources revealed a metabolic gradient spanning fermentative anabolism under glucose, redox adaptation during xylose utilization, and oxidative catabolism during growth on glycerol^17^. Glucose supported growth promoted biosynthetic and proliferative processes fueled by abundant energy and carbon intermediates, whereas xylose favored redox-balancing mechanisms centered on the pentose phosphate pathway. In contrast, glycerol activated respiratory metabolism, β-oxidation, and the glyoxylate cycle. Together, these profiles define a continuum illustrating the capacity of *L. starkeyi* to dynamically rewire carbon metabolism in response to substrate availability, highlighting the coordinated interplay between carbon flux, redox regulation, and biosynthetic capacity that underpins its metabolic flexibility.

### Integrated proteo-lipidomic insights into carbon-dependent lipid metabolism in *Lipomyces starkeyi*

A global lipidomic analysis was performed to assess whether *L. starkeyi* exhibits distinct lipid signatures in response to carbon source variation or nitrogen limitation. Principal component analysis (PCA) of samples cultivated on glucose, glycerol, and xylose under both basal and nitrogen-limited media showed strong overlap among replicates, indicating that the overall lipidomic landscape remained broadly conserved across conditions (Supplementary Fig. S4). Consistently, pairwise volcano plot comparisons revealed a limited number of differentially abundant lipid features, underscoring the subtle extent of lipid remodeling. Glucose versus glycerol comparisons (in both base and nitrogen limited media) yielded four features commonly regulated and thirteen unique to each condition; glucose versus xylose identified one shared and six unique; and xylose versus glycerol presented two shared and eighteen unique differentially abundant species (Supplementary Fig. S4). Within the same carbon source, nitrogen limitation had minimal impact: glucose versus glucose in nitrogen limited media showed no significant alterations, and xylose versus xylose in nitrogen limited media displayed only two downregulated features. The exception was glycerol, which exhibited a broader downregulation of membrane-associated lipids predominantly phosphatidylethanolamines (PE), phosphatidylcholines (PC), and phosphatidylserines (PS) which are structural membrane lipids consistent with a potential reduction in membrane biogenesis or remodeling under nutrient stress^17–21^(Supplementary Fig. S5).

**Fig. 3:**
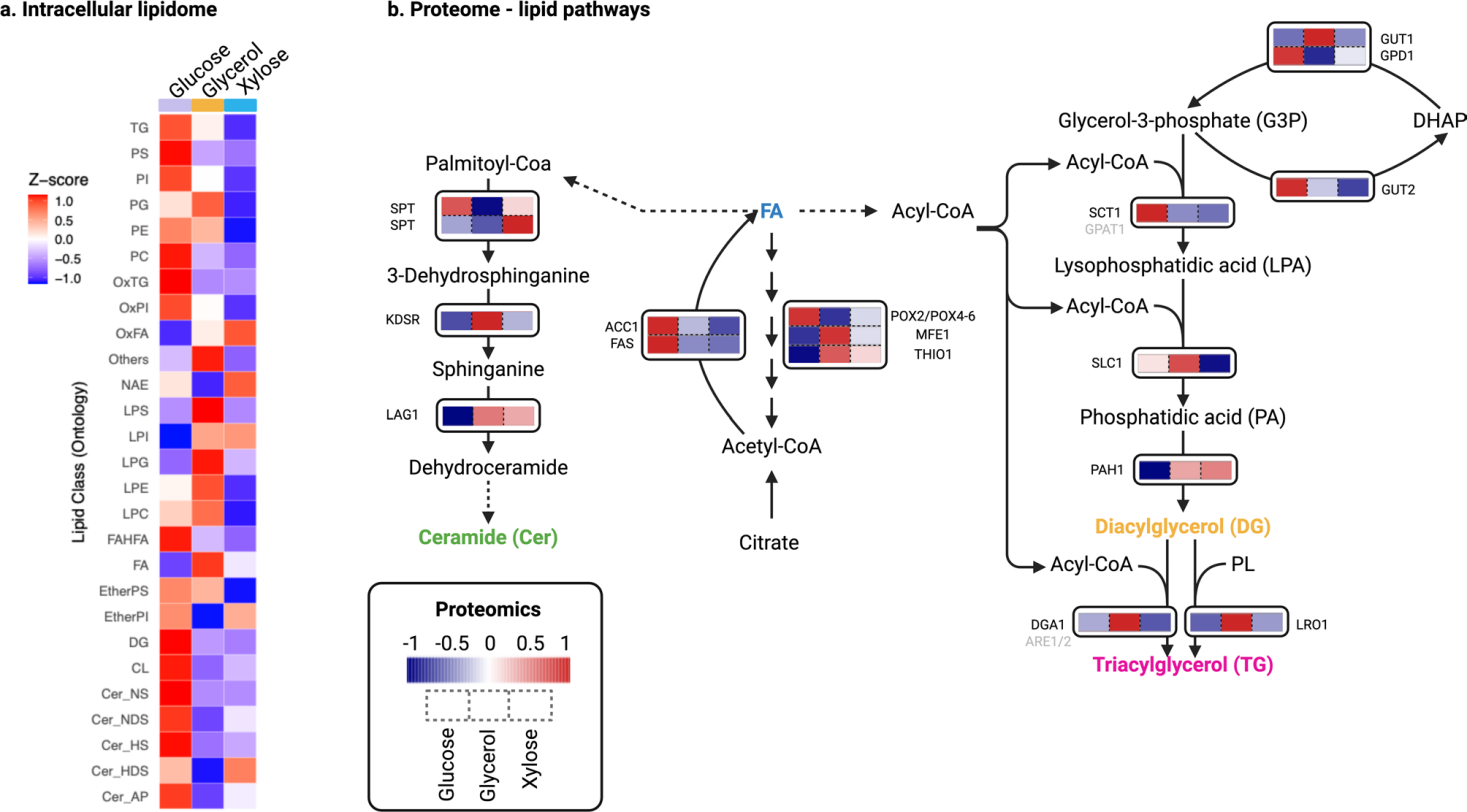
Carbon source–dependent remodeling of lipid composition and lipid-associated enzymes. (**a**) Lipidomics profiling showing relative abundance patterns of major lipid classes across glucose, glycerol, and xylose growth conditions. Lipid species are grouped by class/ontology, highlighting carbon source–specific shifts in storage lipids, membrane lipids, and sphingolipid-related pools. (**b**) Proteomic heatmaps of enzymes involved in lipid metabolic pathways, including fatty acid metabolism, glycerolipid remodeling, and triacylglycerol (TAG) biosynthesis. Enzyme abundance patterns are shown for glucose, glycerol, and xylose conditions, illustrating carbon source–dependent regulation of lipid-associated metabolic capacity. Color scales represent row-wise z-score–scaled group means. Created in BioRender. Oliveira Monteiro, L. M. (2026) https://BioRender.com/h02wf2j.

To identify coordinated compositional trends beyond individual molecular species, lipid features were grouped into ontology-based classes, allowing comparison of broader distribution patterns across carbon sources (Supplementary Fig. S6) and nutrient regimes (Supplementary Fig. S7). The class-level profiles, visualized as normalized Z-score heatmaps (Fig. 3a; Supplementary Figs. S8–S9a), revealed coherent yet substrate-specific shifts in lipid abundance, indicating that carbon source exerts a strong influence on lipidome organization.

To refine these observations, we examined how specific lipid subclasses responded to variation in carbon source and nitrogen availability and integrated these patterns with proteomic data from enzymes involved in fatty acid biosynthesis, elongation, membrane lipid synthesis, and storage lipid metabolism (Fig. 3b; Supplementary Fig. S9b). This proteo-lipidomic integration revealed distinct lipid remodeling strategies across glucose, xylose, and glycerol conditions, linking compositional changes in membrane and storage lipids to corresponding adjustments in lipid metabolic capacity.

Under glucose-supported growth, enzymes central to de novo fatty acid biosynthesis such as acetyl-CoA carboxylase (ACC1) and fatty acid synthase (FAS) were more abundant, consistent with elevated levels of phospholipid classes associated with rapid membrane biogenesis, including phosphatidylcholine (PC) and phosphatidylethanolamine (PE) (Fig. 3 and Supplementary Fig. S8). Glucose also favored the accumulation of cardiolipins (CL), particularly CL 72:9 and 72:10, suggesting increased mitochondrial activity and respiratory metabolism. The enrichment of ceramides further indicates activation of membrane remodeling and oxidative stress response pathways under these highly respiratory conditions (Fig. 3a and Figs. S6 and S8).

In contrast, cultures grown on glycerol displayed increased abundance of diacylglycerol acyltransferase (DGA1) and the glycerolipid remodeling enzyme LRO1, supporting an enhanced enzymatic capacity for triacylglycerol (TAG) synthesis and lipid turnover (Fig. 3b). However, the lipidomic profiles revealed higher accumulation of TAG species under glucose, consistent with the stronger carbon surplus and fermentative overflow characteristic of this condition (Fig. 3). Together, these data suggest that while glycerol promotes the upregulation of TAG-associated enzymes, glucose provides the metabolic environment that favors the actual accumulation of storage lipids. Under nitrogen limitation, *L. starkeyi* exhibited a metabolic transition characterized by reduced levels of unsaturated fatty acids and phospholipids, and by the accumulation of more saturated lipid species typical of stable TAG storage (Figs. S8 and S9). This trend aligns with a transition toward energy preservation and lipid reservoir formation under nutrient stress.

Xylose-grown cultures, on the other hand, showed a reduction in phosphatidylglycerol (PG) and cardiolipin (CL) abundance, indicative of attenuated mitochondrial biogenesis and altered energy metabolism under this less favorable substrate. Together, these patterns suggest that carbon source availability exerts a central role in modulating the balance between membrane lipid biosynthesis, storage lipid accumulation, and lipid turnover in *L. starkeyi*. Glucose supports anabolic lipid synthesis and mitochondrial expansion, whereas glycerol and xylose promote a metabolic reallocation favoring energy conservation and membrane maintenance reflecting adaptive optimization of lipid metabolism across distinct carbon and nitrogen regimes.

### Post-translational modification (PTMs) insights into cellular states

PTMs have an important role in protein structural and regulation, however their exact effects on proteins are often hard to predict and quantify^22^. We collected both REDOX and phosphorylation data here and quantified 5,191 unique oxidized cysteine sites on 2382 proteins across all conditions, similar to that reported for *R. toruloides*^14^. Our phosphoproteomic analysis detected 3,698 unique modified sites corresponding to 3000 unique proteins, much lower than that of *R. toruloides*. We compared cellular redox states and our data indicated that glycerol induced a state of oxidation compared to glucose with 1379 cysteine sites more oxidized at the early timepoint versus 314 sites less oxidized in nitrogen replete conditions (sites with adjusted-p-values < 0.05 and log2FC > 0.38). Meanwhile, xylose had a similar oxidative state as glucose (Fig. S9).

These observations suggest that glycerol metabolism imposes a distinct redox burden relative to fermentative carbon sources, consistent with its stronger reliance on respiratory metabolism and mitochondrial activity. In the previous *R. toruloides* study there was a large, consistent shift in oxidative states between nitrogen replete and deplete conditions^14^. We observed a shift in oxidative states that was dependent on the carbon source. Glucose and xylose nitrogen depleted conditions both showed more reduced redox states while glycerol depleted showed more oxidized redox states compared to their respective replete conditions (Fig. S9).

A similar trend was detected in our phosphoproteomic samples. Glycerol had the largest differential changes in phosphoproteome compared to glucose, with more significantly different sites changed compared to the redox states. More phosphorylated sites showed decreased signals in glucose nitrogen deplete conditions vs. the nitrogen replete control as opposed to the glycerol deplete vs. replete conditions (Fig. S10).

These patters indicate that carbon source not only reshapes protein abundances but also rewires post-translational regulatory landscapes, with glycerol promoting a PTM profile consistent with enhanced oxidative metabolism and altered signaling demands. We next sought to identify the key regulatory proteins driving the molecular responses across carbon sources and through protein abundance and PTM changes.

### Regulatory responses and mechanisms of central metabolism

**Fig. 4:**
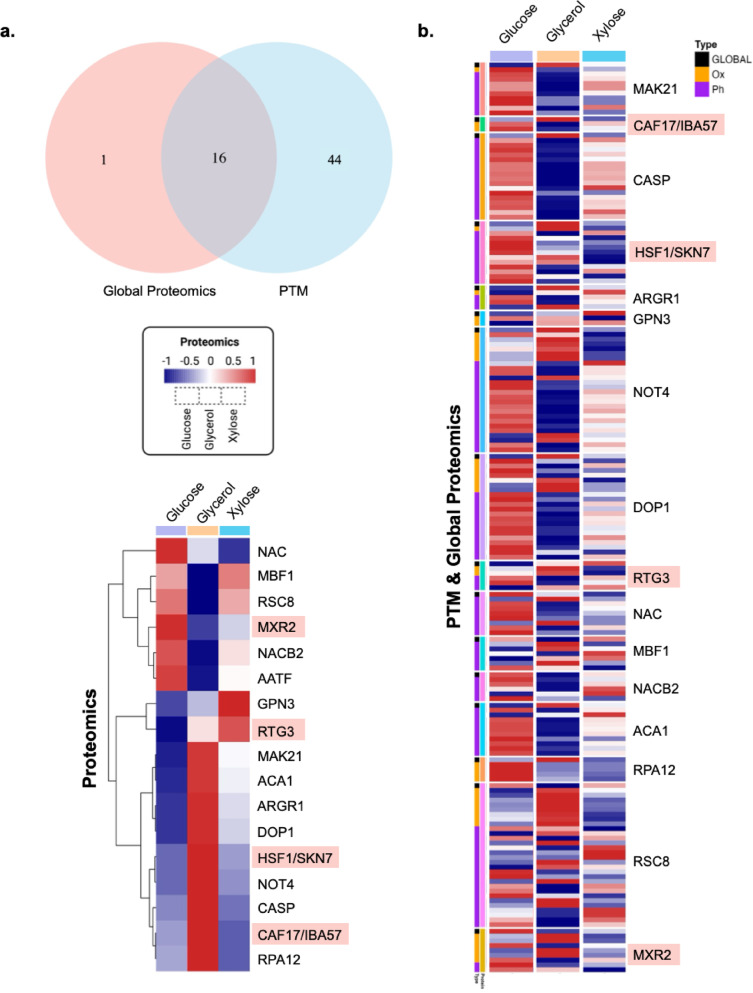
Regulatory proteins identified across global proteomics and post-translational modification (PTM) datasets under different carbon sources. (**a**) Venn diagram showing the overlap between regulatory proteins (RP) detected in global proteomics (*n* = 17) and those identified in the PTM-enriched dataset (*n* = 16). Heatmap of RP abundance profiles derived from global proteomics across glucose, glycerol, and xylose conditions. (**b**) Heatmap of PTM signals for the overlapping RP, displaying relative modification levels across carbon sources, with annotations indicating PTM type (phosphorylation or cysteine oxidation). Highlighted RP represent regulators discussed in the subsequent section that are predicted to interact with enzymes from central carbon metabolism, including the TCA cycle, glycolysis, and lipid metabolic pathways. Color scales represent row-wise z-score–scaled group means. *Abbreviations: ACA1 = ATF/CREB activator 1*,* AATF = Apoptosis antagonizing transcription factor/protein transport protein*,* ARGR1 = Arginine metabolism regulation protein*,* CAF17/IBA57 = Iron-sulfur cluster assembly factor*,* CASP = Protein CASP*,* DOP1 = Protein DOP1*,* GPN3 = GPN-loop GTPase 3*,* HSF1/SKN7 = Heat shock transcription factor*,* MAK21 = Ribosome biogenesis protein*,* MBF1 = Multiprotein-bridging factor 1*,* MXR2 = Peptide methionine sulfoxide reductase*,* NAC = Nascent polypeptide-associated complex*,* NACB2 = Nascent polypeptide-associated complex subunit beta-2*,* NOT4 = General negative regulator of transcription subunit 4*,* RPA12 = DNA-directed RNA polymerase I subunit*,* RSC8 = Chromatin structure-remodeling complex protein*,* RTG3 = Retrograde regulation protein 3.*

The strong carbon source–dependent organization observed in the proteome and lipidome prompted us to examine whether regulatory proteins exhibit similarly structured responses. We therefore focused on proteins annotated with regulatory functions, including transcription factors and transcription factor–like proteins, to assess how regulatory landscapes shift across carbon sources and nitrogen regimes. From the global proteomic dataset, we identified 17 regulatory proteins (RP) that were consistently detected across conditions (Fig. 4a). Analysis of their abundance patterns allowed us to evaluate whether regulatory programs align with the carbon-driven metabolic states observed in central carbon and lipid metabolism. These TFs are generally expressed under conditions that demand tight control of transcriptional and translational processes, such as oxidative metabolism, nutrient limitation, and the transition between fermentative and respiratory growth^23–26^.

Regulatory proteins displayed distinct abundance profiles that clustered primarily according to the carbon source (Fig. 4), with substantial protein abundance differences among glucose-, glycerol-, and xylose-grown cultures that exceeded the effects of nitrogen limitation (Fig. S12). Across all groups, nitrogen limitation modulated RP abundance by typically amplifying or dampening signal intensity but did not fundamentally alter the clustering pattern defined by the carbon source. In other words, samples cultivated with or without nitrogen limitation under the same carbon source maintained closely related signatures, indicating that carbon availability was the primary driver of RP abundance at the early stage analyzed (Fig. S12). These observations are consistent with global multi-omic analyses, in which proteomics and lipidomics data also showed stronger effects driven by carbon source than by nitrogen limitation.

**Fig. 5:**
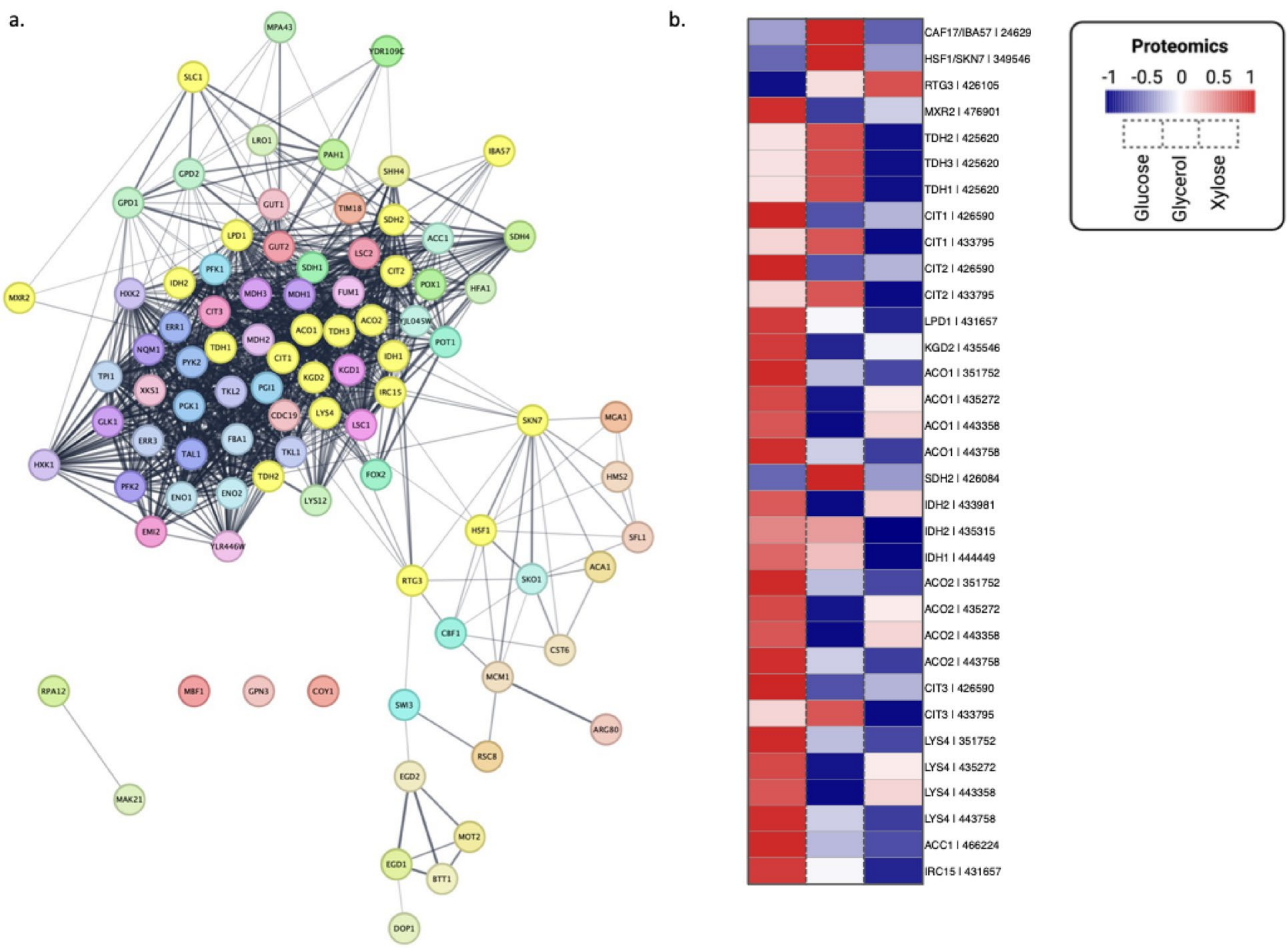
Network-based identification of regulatory proteins interfacing with central carbon and lipid metabolism. (**a**) Protein–protein interaction (PPI) network generated using STRING, integrating regulatory proteins (RP) and metabolic enzymes identified in the proteomics dataset. Nodes highlighted in yellow indicate RP and their directly connected proteins involved in glycolysis, the tricarboxylic acid (TCA) cycle, and lipid metabolic pathways. Edges represent predicted functional associations based on STRING confidence scores. (**b**) Heatmap showing relative protein abundance patterns of the highlighted RP and associated metabolic enzymes across glucose, glycerol, and xylose conditions. Values represent row-wise z-score–scaled group means. *Abbreviations: ACC1 = Acetyl-CoA carboxylase*,* ACO1 = Aconitate hydratase*,* ACO2 = Homocitrate dehydratase*,* CAF17/IBA57 = Iron-sulfur cluster assembly factor*,* CIT1 = Citrate synthase*,* CIT2 = Citrate synthase*,* CIT3 = Citrate synthase 3*,* HSF1/SKN7 = Heat shock transcription factor*,* IDH1-2 = Isocitrate dehydrogenase*,* IRC15 = Increased recombination centers protein 15*,* KGD2 = Dihydrolipoyllysine-residue succinyltransferase*,* LPD1 = Dihydrolipoyl dehydrogenase*,* mitochondrial*,* LYS4 = Homoaconitase*,* MXR2 = Peptide methionine sulfoxide reductase*,* RTG3 = Retrograde regulation protein 3*,* SDH2 = Succinate dehydrogenase*,* TDH1-3 = Glyceraldehyde-3-phosphate dehydrogenase 1–3.*

Hierarchical clustering of regulatory proteins revealed three main groups with contrasting abundance trends. Under glycerol conditions, transcriptional factors linked to stress and transcriptional machinery components (HSF/SKN7) were more abundant (Fig. 4). RPA12, a Pol I subunit that interacts with stress regulators, also showed differential patterns consistent with altered lipid metabolism regulation. CAF17 involved in mitochondrial Fe‑S cluster assembly appeared in interaction networks (Fig. 5), suggesting indirect connections to transcriptional adaptation rather than direct DNA‑binding activity. These profiles likely reflect enhanced transcriptional and translational adjustments required for oxidative and respiratory growth, lipid remodeling, and redox balance during respiratory metabolism consistent with the observed increase in lipid-associated proteins and TCA cycle enzymes under these conditions.

From a functional standpoint, RP predominantly enriched in glycerol (HSF/SKN7, RPA12, CAF17, CASP, DOP1, and ACA1) likely promote transcriptional efficiency, protein homeostasis, and lipid- or redox-associated remodeling characteristic of respiratory metabolism^27–31^. Those enriched under glucose and xylose (MBF1, NAC, NACB2, and GPN3) appear to coordinate translational adaptation, transcriptional coactivation, and stress mitigation pathways, supporting either high biosynthetic flux (glucose) or non-preferred carbon metabolism (xylose).

Of the 17 regulatory proteins identified in the global proteome, 16 were also detected in the post-translational modification (PTM) enriched datasets (cysteine oxidation and phosphorylation), often with distinct modification profiles across carbon and nitrogen conditions (Fig. 4b). Moreover, 44 additional RP were exclusively detected in the PTM datasets, likely reflecting lower abundance in the global proteome yet increased detectability due to enrichment sensitivity (Fig. S11). While PTM data were systematically collected in this study, a detailed mechanistic dissection of individual modification sites and their structural consequences was beyond the scope of the present work. Accordingly, functional interpretation of these modifications remains speculative, the prevalence of PTMs across the RP landscape underscores an additional regulatory layer acting on regulation. Such modifications may fine-tune RP stability, DNA-binding affinity, or redox responsiveness particularly under oxidative or nitrogen-limited conditions that demand dynamic regulatory flexibility^32,33^. These observations reinforce the view that regulation in this yeast operates through multi-level control, integrating expression changes, protein–protein interactions, and structural modulation via PTMs.

To strengthen these interpretations, protein–protein interaction analysis using STRING^34^, along with co-expression analyses, were applied to connect these RP to specific metabolic modules, including central carbon metabolism (glycolysis, TCA cycle, and pentose phosphate pathway) and lipid-related processes. These observations are further discussed in the following sections.

### Regulatory mechanisms linking regulatory proteins to central metabolism

To place these regulatory proteins within a metabolic context, we next examined their predicted interaction networks. Protein–protein interaction analysis using STRING^34^, combined with co-expression patterns, was used to evaluate how regulatory proteins interface with central carbon metabolism and lipid-associated pathways. This network-based perspective enabled identification of regulatory proteins positioned at key interfaces between metabolic enzymes and stress-responsive or transcriptional control processes, providing insight into how carbon source–dependent metabolic states may be coordinated at the regulatory level.

Network analysis using STRING^34^ revealed a subset of regulatory proteins positioned at the interface between central metabolism, lipid pathways, and stress-responsive signaling (Fig. 5). Rather than representing direct transcriptional control in all cases, these regulatory proteins form interaction hubs linking metabolic enzymes with proteins involved in transcriptional, post-transcriptional, and stress-associated regulatory processes.

Among these, RTG3 and SKN7 emerged as prominent and well-supported coordinators of metabolic adaptation across carbon sources. RTG3, a helix–loop–helix transcription factor enriched under xylose conditions, is a central component of the retrograde signaling pathway, which communicates mitochondrial functional status to the nucleus^35,36^. Its predicted connectivity to TCA cycle enzymes, including isocitrate dehydrogenase (IDH1-2), citrate synthase (CIT1-3), and aconitase (ACO1-2), places RTG3 at a strategic regulatory node linking mitochondrial metabolism to transcriptional responses. Although these enzymatic targets were more abundant under glucose and glycerol, the elevated abundance of RTG3 under xylose suggests a compensatory regulatory response aimed at sustaining respiratory capacity when carbon flux through the TCA cycle is diminished.

Similarly, the heat shock transcription factor SKN7 was preferentially enriched in glycerol-grown cultures, particularly under nitrogen limitation, consistent with its established role in oxidative, osmotic, and environmental stress responses^27,37–39^. SKN7 occupied a central position in the network through predicted interactions with aconitase (ACO1-2), dihydrolipoyl dehydrogenase (LPD1), and glyceraldehyde-3-phosphate dehydrogenase (TDH1-3), linking stress-responsive transcriptional regulation to glycolytic and TCA-associated processes. This topology supports a model in which SKN7 integrates redox and stress signals with central carbon metabolism during respiratory growth.

Beyond these canonical regulatory proteins, the network also highlighted regulatory-associated proteins that may modulate metabolism indirectly. A pilin-like regulatory protein annotated as a methionine-R-sulfoxide reductase, enriched under glycerol and nutrient-limited conditions, interacted with enzymes involved in lipid biosynthesis (SLC1) and the TCA cycle (LPD1). While not a classical DNA-binding transcription factor, its position within the interaction network suggests a role in coordinating redox homeostasis and metabolic function under respiratory growth. Likewise, CAF17, a component of the CCR4 complex, is enriched in glycerol conditions and connects to TCA‑associated enzymes in the network. Though not a transcription factor, its role as part of a global gene expression regulator that influences mRNA turnover and translation is consistent with modulation of central metabolic gene expression at post‑transcriptional steps^40–43^.

Together, this regulatory network architecture highlights key interfaces rather than exhaustive mechanisms, emphasizing how carbon source and nutrient availability shape transcriptional and post-transcriptional coordination of central metabolism. Glycerol-driven respiratory growth favors regulatory programs integrating stress tolerance, redox balance, and lipid-associated metabolism, whereas xylose growth activates compensatory regulatory responses aimed at maintaining mitochondrial function. Nitrogen limitation modulates the strength of these responses but does not fundamentally alter their direction, reinforcing carbon source as the dominant driver of regulatory architecture.

## Conclusion

This study demonstrates that carbon source availability acts as a dominant organizing principle of cellular phenotype in the oleaginous yeast *Lipomyces starkeyi*, shaping metabolic flux distribution, lipid allocation strategies, and regulatory network architecture across multiple molecular layers. By integrating proteomics, lipidomics, metabolomics, and post-translational modification profiling, we reveal that phenotypic states associated with fermentative growth, mixed redox metabolism, or oxidative respiration are not defined by single pathways, but instead emerge from coordinated reprogramming of metabolism and regulation.

Beyond cataloging pathway changes, our results highlight how regulatory proteins interface with central carbon and lipid metabolism to encode condition-specific phenotypic programs. The alignment between regulatory proteins abundance, protein–protein interaction topology, and metabolic enzyme expression underscores a systems-level regulatory logic in which regulatory control constrains metabolic behavior. Such multi-layered control provides a mechanistic basis for phenotypic plasticity in response to substrate identity and nutrient availability.

Importantly, these findings advance the interpretation of multi-omic data as a framework for phenotype prediction, rather than descriptive profiling alone. The observed correspondence between carbon source, regulatory state, and metabolic output suggests that integrating regulatory network context with metabolic signatures can improve inference of growth mode, and lipid storage capacity. This perspective aligns with recent multi-omics studies demonstrating that coordinated shifts across proteomic, lipidomic, and metabolomic layers capture fundamental principles governing stress adaptation and carbon fate in eukaryotic microbes^19^.

From an applied standpoint, this work provides critical insight for advancing metabolic engineering and bioproduction in oleaginous yeasts. Understanding how specific carbon sources impose distinct regulatory and metabolic constraints enables more informed strategies for strain design, pathway optimization, and process control. By linking regulatory architecture to metabolic phenotype, our study establishes a foundation for predictive models that can guide rational engineering of lipid accumulation, redox balance, and carbon utilization efficiency in *L. starkeyi* and related biomanufacturing chassis.

Beyond systems-level characterization, our integrated multi-omic analysis highlights several candidate targets for metabolic engineering aimed at enhancing lipid accumulation in *L. starkeyi*. Key enzymes involved in triacylglycerol synthesis, such as DGA1 and LRO1, emerged as central nodes associated with lipid storage capacity. In addition, the coordinated regulation of β-oxidation and acetyl-CoA metabolism suggests that modulating fatty acid degradation pathways may represent a strategy to optimize carbon flux toward lipid biosynthesis. Transcriptional regulators, including RTG3 and SKN7, were also identified as potential control points linking metabolic state, redox balance, and lipid metabolism. Together, these findings provide a framework for rational strain engineering and highlight the value of multi-omic approaches in identifying actionable targets in non-model oleaginous yeasts.

## Materials and methods

### Chemicals, strains, and growth conditions

Chemicals used for routine culturing were purchased from Sigma-Aldrich (St. Louis, MO) unless noted otherwise. *L. starkeyi* strain NRRL Y-11,558 was obtained from American Type Tissue Culture (ATCC64135; Manassas, VA). The strain was recovered from glycerol stocks on yeast-peptone-dextrose (YPD) agar plates containing 10 g/L yeast extract, 20 g/L peptone, 20 g/L dextrose, 15 g/L agar. Plates were grown for 5 days at 28 C. Five colonies were then picked and inoculated into 2 mL of YPD media (same formula as YPD agar without agar) and grown for 2 days at 28 C. Strains were then transferred to yeast-nitrogen-base (YNB) without amino acids or nitrogen source and grown for two days as seed cultures. The seed culture was used to inoculate YNB media containing 40 g/L of the specified carbon source and either ammonium chloride or urea at a carbon to nitrogen ratio of ~ 17 or ~ 80. Initial pH was adjusted to 5.6. Cultures were grown in shaking flasks at 28 °C and 200 rpm under aerobic conditions. These conditions are expected to provide sufficient oxygen transfer to support respiratory metabolism, particularly during growth on non-fermentable carbon sources such as glycerol.

### FAME and extracellular metabolite analysis

Two aliquots of 10–15 mL of cell culture were collected for each individual omic analysis pipelines (proteomics/PTMs & metabolomics/lipidomics). Cells were pelleted via centrifugation for 5 min at 4,000 x g and 4 C, the supernatant was discarded, and the cells were washed once with PBS before being frozen in liquid nitrogen. For extracellular metabolite analysis, the supernatant/culture broth was filtered through 0.22 μm filters and frozen until analysis was conducted. For dry cell weight and FAME determination, 10–15 mL of remaining culture was pelleted in a pre-weighed conical tube (5 min at 4,000 x g), washed once with PBS, and frozen at -20 °C. The frozen pellet was then lyophilized for 24–36 h.

The esterification process consisted of weighing about 10–20 mg of lyophilized cell pellet and transferred to a screw cap microtube where the cells were lysed via bead beating. The samples were then mixed with three reagents in the following order: 100 µL of 10 mg/mL methyl tridecanoate in methanol, 750 µL of 3 M methanolic hydrogen chloride, and 50 µL of chloroform. The mixture was bead beaten and incubated for an hour at 80 °C followed by another round of bead beating. To extract the FAMEs, 500 µL of n-hexanes was added to the samples and thoroughly mixed and spun down (10,000 x g for 1 min) to separate the layers. The organic layer was then transferred to 1.5 mL vials for GC analysis.

### Intracellular lipidomic and metabolomics sample preparation

Metabolites and lipids were extracted using a modified Folch extraction called MPLEx^44,45^. A solution of -20˚C, chloroform: methanol (2:1, v/v) was added to each sample for a final ratio of chloroform: methanol: water of 8:4:3, v/v/v. Samples were vortexed for 1 min, incubated on ice for 5 min, and then vortexed again for 1 min. Samples were centrifuged at 10,000 x g for 10 min at 4˚C. The lower organic layer was removed, dried in vacuo then stored at -20˚C in chloroform: methanol (2:1, v/v) until lipidomic analyses. Just prior to lipidomic analysis, total lipid extracts (TLEs) are reconstituted in 200uL of 10% chloroform and 90% methanol. Samples with precipitate in the high methanol mixture and were spun down at 4500 rpm for 5 min before the supernatant was transferred to a new vial, which was repeated two separate times. For metabolomic analysis, the methanol organic layer was removed with part of the lower (chloroform) layer added. In vacuo drying was used followed by a resuspension in 80:20 MeOH: H2O (4:1, v/v). To each of the original vials, 100 uL of 80/20 MeOH/H2O was added and sonicated for 10 min. Samples were then vortexed followed by centrifuging at 20 x g for 10 min. A 0.2 μm PTFE 96 filter well plate was conditioned with 1 mL methanol followed by 1 mL Milli-Q H2O. N2 pushed the sample through the well plate into a corresponding vial which was stored at -20˚C in 80:20 MeOH: H2O (4:1, v/v) until MS analysis.

### Metabolomics separation and mass spectrometry data collection

For reverse phase liquid chromatography (LC), the mass spec method (see below) was set for a 10 min acquisition. Waters H-Class LC was paired to the mass spectrometer. The column used consisted of a Waters Acquity BEH 1.7 μm, 2.1 mm x 100 mm length paired with a Vanguard pre-column with the same packing material held at a temperature of 40 °C. An injection volume of 5uL was used for both modes. All solvents and additives used were LC-MS grade. Reverse phase positive mode mobile phase A (MPA) consisted of 0.1% formic acid in Milli-Q H2O with mobile phase B (MPB) made up of 0.1% formic acid in methanol. Reverse phase negative mode MPA consisted of 6.5 mM ammonium bicarbonate in water at a pH of 8 with MPB made up with 6.5 mM ammonium bicarbonate in 95% methanol and 5% Milli-Q H2O. The gradient was held at 0.35 mL/min consisting of 99.5% MPA at 0 min, 30% MPA at 4 min, 2% MPA from 4.5 to 5.4 min, and 99.5% MPA from minute 5.6 through minute 15. This was used for both polarities. Note that MPB makes up the remaining percents and minutes 10 to 15 are for washing and equilibration and are not acquired by the Q-Exactive.

For HILIC phase, the mass spec method (see below) was set for a 16 min acquisition with the settings listed above. A Waters H-Class LC was paired to the mass spectrometer. The column used consisted of a Waters Acquity BEH Amide 1.7 μm, 2.1 mm x 150 mm length paired with a Vanguard pre-column with the same packing material held at a temperature of 40 °C. An injection volume of 3uL was used for both modes. All solvents and additives used were LC-MS grade. HILIC MPA consisted of 0.125% formic acid and 10 mM ammonium formate in Milli-Q H2O with mobile phase B MPB made up of 0.125% formic acid and 10 mM ammonium formate in in 95% acetonitrile and 5% Milli-Q H2O. The same solvents were used for positive and negative modes. The gradient was held at 0.4 mL/min consisting of 0% MPA from 0 to 2 min, 30% MPA from 5 to 5.7 min, 60% MPA from 7 to 7.5 min, 70% MPA at 8.25 min, and 100% from 10.75 to 21 min. This was used for both polarities. Note that MPB makes up the remaining percents and minutes 16 to 21 are for washing and equilibration and are not acquired by the Q-Exactive.

Samples are analyzed on a Thermo Q-Exactive Plus in both positive and negative ionization modes using higher-energy collision dissociation (HCD). A heated electrospray ionization (HESI) source was used with a spray voltage 4 kV with a capillary temperature of 250 °C and a sheath gas temperature of 200 °C. Datasets were collected on a rate of 2 spectra per second. Full MS scans were acquired at a resolving power of 70,000 FWHM at m/z 200 with a scanning range of m/z 80–1000. The automatic gain control (AGC) target was set at 3E6 ions, with a maximum injection time of 20 ms. The data dependent acquisition (dd-MS2) parameters used to obtain product ion spectra were as follows: resolving power 17,500 FWHM at m/z 200, AGC target 1E5 ions with maximum injection time of 100 ms, isolation width 1.0 m/z, loop count 4, and normalized collision energy (NCE): 20, 30, 40 eV.

### Lipidomic separation and mass spectrometry data collection

TLEs are analyzed using liquid chromatography electrospray ionization tandem mass spectrometry (LC-ESI-MS/MS) comprised of a Thermo Vanquish Flex system interfaced with a Thermo Lumos mass spectrometer. The HESI source parameters are set as follows: spray voltage 3.5 or 3.4 kV for positive and negative modes respectively; capillary temperature 350 °C; S lens RF level 30 arbitrary units; aux gas heater temperature 350 °C; sheath, auxiliary, and sweep gas flows of 50, 10, and 1, respectively. Full MS scans were acquired at a resolving power of 120,000 FWHM at m/z 200 with the scanning range of m/z 200–1800. The data dependent acquisition (dd-MS2) parameters used to obtain product ion spectra are as follows: time limit of 1s with scans alternating between collision-induced dissociation (CID) and HCD, isolation width of 2 m/z units, default charge state of 1, activation Q value of 0.18 for CID, HCD resolving power of 7,500 FWHM at m/z 200, normalized collision energies for CID of 38 with detection in the ion trap and stepped collision energy of 25, 30, and 35 for HCD with detection in the orbitrap. The mass spec method was set for a 25 min acquisition for both polarities.

The column used was a Waters charged surface hybrid (CSH) 1.7 μm, 3 mm x 150 mm length paired with a Vanguard pre-column with the same packing material held at a temperature of 50 °C. An injection volume of 5 uL was used for positive mode and a 10 uL injection was used for negative mode. All solvents and additives used were LC-MS grade. MPA consisted of 10 mM ammonium acetate in 60% Milli-Q H2O and 40% acetonitrile. MPB consisted of 10 mM ammonium acetate in 90% isopropanol and 10% acetonitrile. The same solvents were used for both modes with the full amount needed in each mode being mixed beforehand to reduce lipid retention time shifts. The gradient was held at 0.3 mL/min consisting of 60% MPA at 0 min, 38% MPA at 1 min, 34% MPA at 4 min, 22% MPA at 9 min, 13% MPA at 11 min, 1% MPA from 15 to 21 min, 60% MPA at 21.1 min, 1% MPA at 21.6 min, and 60% MPA from 21.7 to 29 min. Note that MPB makes up the remaining percents and minutes 25 to 29 are for washing and equilibration and are not acquired by the Lumos.

### Metabolomic and lipidomic data processing

Metabolite and lipid identifications were made using MS-DIAL v4.92 for peak detection^46^, identification and alignment. The experimental data was matched both to in-house libraries (m/z less than 0.003 Da, retention time less than 0.3 min, MS/MS spectral match) and a compilation of publicly available MS/MS databases (m/z less than 0.003 Da, MS/MS spectral match) (VS17) available in MS-DIAL. The tandem mass spectra and corresponding fragment ions, mass measurement error, and aligned chromatographic features were manually examined to remove false positives. Relative quantification was performed by calculating peak areas on extracted ion chromatograms of precursor masses.

### Proteomic and post-transcriptional modification separation and mass spectrometry data collection

Global protein abundance and PTM (i.e., redox and phosphorylation) profiling was performed using an established method^14,47^. Briefly, cell pellets were incubated and lysed via bead beating in 250 mM MES (pH 6.0), 5% SDS, and 1% Triton X- 100 with 100 mM N-ethylmaleimide (NEM). Cell lysates containing 1 mg protein from each sample were transferred into a KingFisher 96 deep-well plate (sample/binding plate) as input for automated SP3 sample cleanup and digestion. Sample volume was adjusted to 500 µl with lysis buffer as mentioned above, and 500 µl absolute ethanol was added into each well. SP3 beads (of a weight ratio of protein: bead = 1:5) were washed and transferred to a bead plate. Three wash plates were prepared containing 1 mL 80% ethanol. All plates were loaded onto KingFisher Flex (Thermo Scientific, CA), and the modified R2-P1 program was performed. The digestion was conducted offline using 1:100 trypsin and 1:100 Lys-C for 3 h at 37 °C with 500 rpm shaking on a ThermoMixer Thermo Scientific). The digested peptides were eluted with 250 mM HEPES (pH 7.7) with 5% acetonitrile (ACN) on a MagnaBot FLEX 96 magnetic plate (Promega). For multiplexed quantification, 200 µg peptides from each sample were labeled with tandem mass tags (TMTpro 18-plex, Thermo Scientific) in a ratio of 2.5:1 (TMT: peptide, w/w). TMT-labeled samples were desalted and split for global proteomics quantification and PTM enrichment. Resin-assisted capture (RAC) of oxidized cysteine-containing peptides were performed as described^48^. Six fractions of global proteomics and redox proteomics were generated respectively by high-pH reverse phase capillary LC fractionation. Phosphopeptide enrichment was performed using immobilized metal affinity chromatography (IMAC) as previously described^49^. Six.

fractions of phosphoproteomics were generated. All samples were analyzed by a Vanquish Neo UHPLC (Thermo Scientific) coupled to an Orbitrap Exploris 480 (Thermo Scientific). In-house packed reverse- hase column (25 cm × 75 μm inner diameter, 1.7-µm Waters BEH C18 particles) were used for 60 min LC gradients. Data-dependent acquisition (DDA) was used. The full MS scan range was 400–2,000 m/z with 60 K resolution. Top 20 most abundant precursors were selected for fragmentation (isolation window of 0.7 m/z) by^46^(collision energy at 30%). MS/MS scans were acquired with 45,000 resolution. Fixed first mass was set to 120 m/z.

### Proteomic and post-transcriptional modification data processing

The LC-MS/MS data were searched using MS-GF+^50^ against with the reporter ion intensities extracted using MASIC (https://github.com/pnnl-comp-mass-spec/MASIC). Peptide spectra data was analyzed with PlexedPiper^51^ for the post-processing of TMT reporter ions to perform isobaric quantification. The peptide spectra matches were filtered using the following criteria: (1) mass accuracy within 10 ppm; (2) PepQ value < 0.01 to a final false-discovery rate < 1%. For phosphoproteomics data, a further Ascore calculation was performed to identify the ambiguous modification sites, where Ascore > 17 is considered confident. These were then processed using a custom pipeline for relative quantification of protein abundance, cysteine oxidation (“Redox”), phosphorylation (“Phospho”). Specifically, all the multi-PTM proteomics data including the companion global proteomics data are feed into the ProteoMeter pipeline^52^. First, for global protein abundance data, peptide TMT reporter ion intensities were aggregated to the unique protein level by summing up raw reporter ion intensities of corresponding peptides. These data were then log2 transformed, batch corrected for multiple plexes, and median centered for normalization. Secondly, for PTM abundance data, peptide TMT reporter ion intensities were aggregated to the single amino acid residue level. The log2-transformed global peptide data (not batch corrected) was used to scale the site level PTM abundances to account for TMT channel loadings and for median centered normalization. These PTM data were then batch corrected. Finally, the average normalized protein abundances (per condition) were subtracted from the corresponding PTM site level abundances. IDs with more than two missing values values per condition (2/4 missing values) were removed prior to statistical tests.

### Data analysis

#### Proteomic data processing

Targeted and Global proteomics analysis were processed using R (v4.1.1). Raw protein abundance tables were first curated to ensure consistent sample naming and correct grouping of biological replicates. For each experimental condition, biological quadruplicates were considered, and proteins were retained for downstream analysis only if they were detected in at least two biological replicates. When required, replicate-level measurements were averaged to obtain a single abundance value per protein and condition. For exploratory data analysis, principal component analysis (PCA) was performed on log-transformed and normalized protein abundance matrices to assess global variance structure and sample clustering across conditions. Prior to PCA, missing values and zeros were handled by replacement with the minimum positive value observed in the dataset, followed by log2 transformation. Pairwise differential abundance analyses were conducted between selected experimental conditions. For each protein, fold change and statistical significance were computed using two-sided Student’s t-tests. Proteins with a p-value < 0.05 were considered significantly different between the two groups. For visualization purposes, log2 fold changes (log2FC) were calculated and used in volcano plots, bar plots, and heatmaps. Volcano plots were generated to simultaneously visualize effect size (log2FC) and statistical significance (− log10 p-value), with thresholds applied to highlight upregulated and downregulated proteins. Functional annotation of proteins was performed by integrating KOG and KEGG databases (annotations obtained from the JGI annotation pipeline (https://mycocosm.jgi.doe.gov/LstY11558_1/LstY11558_1.home.html). Protein identifiers were mapped to KOG functional categories and KEGG orthologs, pathways, modules, and reactions. Annotation tables were merged with the quantitative proteomics data to enable pathway-level interpretation and targeted downstream analyses, including focused examination of metabolic pathways and regulatory protein subsets.

#### Regulatory protein analysis and protein–protein interaction networks

Putative regulatory proteins were identified based on functional annotation and sequence homology. Protein sequences from *L. starkeyi* were mapped to orthologous groups using KOG annotations obtained via UniProt, enabling the identification of homologous regulatory proteins annotated in the model yeast *Saccharomyces cerevisiae*. This homology-based mapping was used to transfer curated functional information from *S. cerevisiae*, including transcriptional regulators and signaling-related proteins, to the non-model organism under study. Proteins annotated as transcription factors or regulatory components based on KOG functional classes and UniProt descriptions were retained for downstream analyses. Protein–protein interaction (PPI) networks were reconstructed using STRING^34^, using *S. cerevisiae* as the reference organism. STRING analyses were restricted to experimentally supported and database-curated interactions to minimize spurious associations. The resulting interaction networks were used to explore connectivity patterns among regulatory proteins and to identify highly connected nodes suggestive of central regulatory roles.

#### Lipidomics and metabolomics data processing

Lipidomics data acquired in positive (POS) and negative (NEG) ionization modes were processed independently using R (v4.1.1). Data from each ionization mode were first loaded separately and then harmonized by aligning lipid identifiers. A custom preprocessing workflow was applied to clean and normalize the data. This procedure separated categorical metadata from numerical intensity values, replaced missing values and zeros with the minimum positive value detected across samples, filtered features with insufficient data coverage, and applied log2 transformation. After mode-specific preprocessing, POS and NEG datasets were combined into a single normalized matrix for downstream analyses. Data were reshaped into long format where required to facilitate statistical testing and visualization. Exploratory PCA was performed to evaluate global lipidomic variation across experimental conditions. For differential abundance analyses, pairwise comparisons between experimental groups were conducted. For each lipid feature, mean abundances were calculated per group, and log2 fold changes were computed. Statistical significance was assessed using two-sided Student’s t-tests, with p-value thresholds of 0.05 used to identify significantly altered lipids. Results from pairwise comparisons were exported as tabulated files and visualized using volcano plots. Hierarchical clustering and heatmap visualizations were generated to examine patterns of lipid abundance across samples and conditions. Heatmaps were constructed using row-wise scaling, and clustering was applied selectively depending on the number of significant features identified. For multi-comparison summaries, selected significant or trending lipid features were visualized across multiple condition pairs in panel heatmaps. To facilitate biological interpretation, lipid features were annotated using curated lipid annotation tables, including ontology information. Normalized lipid abundances were integrated with ontology annotations and visualized using grouped boxplots stratified by experimental condition and lipid class.

#### Data visualization

Data visualizations were performed in R using ggplot2, pheatmap, and gridExtra. Heatmaps display scaled abundances (z-score) per condition. Volcano plots highlight significantly regulated proteins or metabolites based on log2FC and p-value thresholds. Boxplots were used to show the distribution of features within ontology groups. All scripts for normalization, statistical analysis, and plotting were implemented in R (v4.1.1) and are available in the GitHub repository: https://github.com/PNNL-Predictive-Phenomics/Lst-SF-MOAnalysis/tree/main.

## Supplementary Information

Below is the link to the electronic supplementary material.

Supplementary Material 1

## Data Availability

Primary raw mass spectrometry lipidomics and metabolomics data have been deposited at MassIVE under the accession: MSV000100642 [doi:10.25345/C5K35MT3C]. This data can be accessed via: [https://massive.ucsd.edu/ProteoSAFe/dataset.jsp? task=d0a8b954d73f416d841791b1f7ed82a3](https:/massive.ucsd.edu/ProteoSAFe/dataset.jsp? task=d0a8b954d73f416d841791b1f7ed82a3) with User: MSV000100642 and password: czajkalip.Primary raw proteomic and post-translational modification data have been deposited in the MassIVE database under the accession number: MSV000100611. The data can be accessed via: [https://massive.ucsd.edu/ProteoSAFe/dataset.jsp? task=5f3298c23818410f82f929ca6772b4cc](https:/massive.ucsd.edu/ProteoSAFe/dataset.jsp? task=5f3298c23818410f82f929ca6772b4cc) with User: MSV000100611 and password: Carbon6510. Code to analyze data have been deposited at the following repository: [https://github.com/PNNL-Predictive-Phenomics/Lst-SF-MOAnalysis.git](https:/github.com/PNNL-Predictive-Phenomics/Lst-SF-MOAnalysis.git) . All other data generated is provided in the manuscript and supplemental files.
