# Supplementary material for: Carbon source–driven metabolic and regulatory remodeling defines phenomic states in Lipomyces starkeyi
Source: Sci Rep. 2026 Aug 12;16:25118. doi: 10.1038/s41598-026-53531-2 (PMC13469140; doi:10.1038/s41598-026-53531-2)
Supplement: Supplementary file 1 [file 41598_2026_53531_MOESM1_ESM.docx]

**SUPPLEMENTARY INFORMATION**

**Carbon source–driven metabolic and regulatory remodeling defines phenomic states in *Lipomyces starkeyi***

Lummy M.O. Monteiro^1^, Xiaolu Li^1^, Kyle R. Pomraning^2^, Jasmin Alvarez^2^, Song Feng^1^, Teresa Lemmon^2^, Marie Swita^2^, Heather Olson1, Josie G Eder^1^, Tong Zhang^1^, Sneha Couvillion^1^, Jason E. McDermott^1,3^, Jeffrey J. Czajka^2,*^

**Affiliations.**

^1^ Earth and Biological Sciences Directorate, Pacific Northwest National Laboratory, Richland, Washington, USA

^2^ Energy and Environment Directorate, Pacific Northwest National Laboratory, Richland, Washington, USA

^3^ Department of Molecular Microbiology and Immunology, Oregon Health & Science University, Portland Oregon

^*^ Correspondence addressed to [Jeffrey.czajka@pnnl.gov](mailto:Jeffrey.czajka@pnnl.gov)

**Table of Contents**

[Supplementary Tables	2](#_Toc219280136)

[Table S1. Phenotypic outcomes of media growth.	2](#_Toc219280137)

[Supplementary Figures	3](#_Toc219280138)

[Fig. S1 Carbon source–dependent proteomic separation under basal and nitrogen-limited conditions.	4](#_Toc219280139)

[Fig. S2 Proteomic impact of nitrogen limitation within each carbon source.	6](#_Toc219280140)

[Fig. S3 Extended proteomic remodeling of central carbon metabolism across carbon and nitrogen conditions.	8](#_Toc219280141)

[Fig. S4 Carbon source–dependent lipidomic separation under basal and nitrogen-limited conditions.	10](#_Toc219280142)

[Fig. S5 Lipid impact of nitrogen limitation within each carbon source.	11](#_Toc219280143)

[Fig. S6 Distribution of lipid features by lipid ontology across carbon sources.	12](#_Toc219280144)

[Fig. S7 Distribution of lipid features by lipid ontology across carbon sources and nitrogen availability.	13](#_Toc219280145)

[Fig. S8 Heatmap analysis of lipidomic features across carbon and nitrogen conditions.	14](#_Toc219280146)

[Fig. S9. Volcano plots of REDOX states	16](#_Toc219280147)

[Fig. S10 Volcano plots of Phosphorylated states	17](#_Toc219280148)

[Fig. S11 Carbon source– and nitrogen-dependent remodeling of lipid composition and lipid-associated enzymes.	18](#_Toc219280149)

[Fig. S12 Top 100 regulatory proteins identified exclusively on post-translational modification (PTM) datasets under different carbon sources.	19](#_Toc219280150)

[Fig. S13 Regulatory proteins identified across global proteomics and post-translational modification (PTM) datasets under different carbon sources and nitrogen availability.	21](#_Toc219280151)

# Supplementary Tables

## Table S1. Phenotypic outcomes of media growth.

| Condition | DCW  (g/L) – Early | Lipid Titer  (g/L) – Early | DCW  (g/L) – Late | Lipid Titer  (g/L) – Late |
| --- | --- | --- | --- | --- |
| YPD | 4.395 ± 1.237 | 0.522 ± 0.441 | 13.310 ± 0.508 | 2.301 ± 0.535 |
| Glucose – C/N 16 | 1.042 ± 0.067 | 0.582 ± 0.850 | 5.452 ± 1.215 | 1.230 ± 0.095 |
| Xylose – C/N 16 | 1.318 ± 0.257 | 0.227 ± 0.012 | 4.473 ± 0.552 | 1.258 ± 0.244 |
| Glycerol – C/N 16 | 1.327 ± 0.415 | 0.218 ± 0.065 | 4.148 ± 1.966 | 1.357 ± 0.624 |
| Glucose+Urea – C/N 16 | 1.087 ± 0.117 | 0.162 ± 0.018 | 2.986 ± 0.035 | 0.455 ± 0.015 |
| Glucose – C/N 80 | 1.235 ± 0.191 | 0.213 ± 0.044 | 5.768 ± 0.913 | 1.813 ± 0.217 |
| Xylose – C/N 80 | 1.198 ± 0.072 | 0.109 ± 0.075 | 5.605 ± 0.149 | 1.338 ± 0.345 |
| Glycerol – C/N 80 | 1.245 ± 0.135 | 0.222 ± 0.022 | 7.585 ± 2.358 | 2.933 ± 1.228 |
| Glucose+Urea – C/N 80 | 0.865 ± 0.091 | 0.186 ± 0.024 | 4.441 ± 0.438 | 1.152 ± 0.157 |

# Supplementary Figures

## Fig. S1 Carbon source–dependent proteomic separation under basal and nitrogen-limited conditions.

Principal component analysis (PCA) of proteomic profiles from *L. starkeyi* grown on glucose, xylose, and glycerol under basal (BASE) medium (left panels) and under basal and nitrogen-limited (NLIM) conditions (right panels). PCA plots illustrate clustering primarily driven by carbon source across conditions. Corresponding volcano plots below each PCA depict pairwise differential protein abundance comparisons between carbon sources under BASE conditions (glucose vs glycerol, glucose vs xylose, glycerol vs xylose; left) and under NLIM conditions (glucose NLIM vs glycerol NLIM, glucose NLIM vs xylose NLIM, glycerol NLIM vs xylose NLIM; right). Proteins were considered significantly regulated using an adjusted p-value < 0.05 and an absolute log₂ fold change ≥ 1. Overlap analysis between BASE and NLIM volcano plots highlights proteins that are commonly upregulated or downregulated between equivalent carbon source comparisons, indicating conserved proteomic responses independent of nitrogen availability.

## Fig. S2 Proteomic impact of nitrogen limitation within each carbon source.

Volcano plots comparing protein abundance between basal (BASE) and nitrogen-limited (NLIM) conditions for each carbon source: glucose (BASE vs NLIM), glycerol (BASE vs NLIM), and xylose (BASE vs NLIM). Proteins were considered significantly regulated using an adjusted p-value < 0.05 and an absolute log₂ fold change ≥ 1. Overlap analysis was performed to identify proteins commonly up- or downregulated across carbon sources under nitrogen limitation, highlighting conserved proteomic responses associated with nitrogen stress independent of carbon source.

Fig. S3 Extended proteomic remodeling of central carbon metabolism across carbon and nitrogen conditions.
Schematic representation of glycolysis, the pentose phosphate pathway (PPP), and the tricarboxylic acid (TCA) cycle highlighting relative protein abundance changes under glucose, xylose, glycerol, glucose NLIM, xylose NLIM, and glycerol NLIM growth conditions. Heatmaps associated with individual enzymatic steps display normalized proteomic abundance patterns across all conditions, illustrating coordinated metabolic shifts driven primarily by carbon source and modulated in magnitude by nitrogen availability. Created in BioRender. Oliveira Monteiro, L. M. (2026) https://BioRender.com/5hat9hg.

## Fig. S4 Carbon source–dependent lipidomic separation under basal and nitrogen-limited conditions.

Principal component analysis (PCA) of lipidomic datasets shows distinct clustering primarily driven by carbon source under basal (BASE) and nitrogen-limited (NLIM) conditions. Volcano plots display pairwise differential lipid abundance comparisons between glucose, glycerol, and xylose under each condition. Lipid features were considered significantly regulated using an adjusted p-value < 0.05 and an absolute log₂ fold change ≥ 0.5. Overlap analysis identifies lipid species commonly altered between BASE and NLIM comparisons, indicating conserved carbon source–specific lipidomic responses and showing that nitrogen limitation modulates, but does not override, carbon-driven lipid remodeling.

## Fig. S5 Lipid impact of nitrogen limitation within each carbon source.

Volcano plots comparing lipid abundance between basal (BASE) and nitrogen-limited (NLIM) conditions for each carbon source: glucose (BASE vs NLIM), glycerol (BASE vs NLIM), and xylose (BASE vs NLIM). Lipid features were considered significantly regulated using an adjusted p-value < 0.05 and an absolute log₂ fold change ≥ 0.5. Overlap analysis was performed to identify lipid species commonly up- or downregulated across carbon sources under nitrogen limitation, highlighting conserved lipidomic signatures associated with nitrogen stress independent of carbon source.

Fig. S6 Distribution of lipid features by lipid ontology across carbon sources.
Boxplots show the abundance distribution of lipid features grouped by lipid class for glucose-, glycerol-, and xylose-grown cultures. Lipid abundances are displayed as log₂(normalized+1), highlighting carbon source–dependent differences in lipid class representation and overall lipid remodeling.

Fig. S7 Distribution of lipid features by lipid ontology across carbon sources and nitrogen availability.
Boxplots show log₂(normalized+1) for glucose, glycerol, and xylose under basal and nitrogen-limited (NLIM) conditions, illustrating how nitrogen limitation modulates lipid class distributions within each carbon source context.

## Fig. S8 Heatmap analysis of lipidomic features across carbon and nitrogen conditions.

Panel of heatmaps representing the relative abundance of lipid features under different growth conditions. **Row 1** compares carbon sources in BASE medium (glucose vs glycerol, glucose vs xylose, and glycerol vs xylose) to identify carbon source–specific lipid markers. **Row 2** shows the same carbon source comparisons in nitrogen-limited (NLIM) medium to evaluate whether these changes are consistent under nitrogen limitation. **Row 3** compares BASE vs NLIM conditions within the same carbon source (glucose, glycerol, and xylose) to assess the effect of nitrogen limitation on lipid composition. Data in each heatmap were normalized per lipid feature (row) using z-score transformation to highlight relative differences

Fig. S9. Volcano plots of REDOX states in (A) Xylose N-replete vs Glucose N-replete, (B) Glycerol N-replete vs Glucose N-replete, (C) Glucose N-deplete vs Glucose N-replete, (D) Xylose N-deplete vs Xylose N-replete, (C) Glycerol N-deplete vs Glycerol N-replete. Red dots represent cysteine sites that are more oxidized when comparing the conditions, with blue being less oxidized sites.

Fig. S10 Volcano plots of Phosphorylated states in (A) Xylose N-replete vs Glucose N-replete, (B) Glycerol N-replete vs Glucose N-replete, (C) Glucose N-deplete vs Glucose N-replete, (D) Xylose N-deplete vs Xylose N-replete, (C) Glycerol N-deplete vs Glycerol N-replete. Red dots represent sites that are more phosphorylated when comparing the conditions, with blue being less phosphorylated sites.

Fig. S11 Carbon source– and nitrogen-dependent remodeling of lipid composition and lipid-associated enzymes. **a.** Lipidomics profiling showing relative abundance patterns of major lipid classes across glucose, glycerol, and xylose growth conditions under basal and nitrogen-limited (NLIM) media. Lipid species are grouped by class/ontology, highlighting carbon source–specific and nitrogen-dependent shifts in storage lipids, membrane lipids, and sphingolipid-associated pools. **b.** Proteomic heatmaps of enzymes involved in lipid metabolic pathways, including fatty acid metabolism, glycerolipid remodeling, and triacylglycerol (TAG) biosynthesis, shown across glucose, glycerol, and xylose conditions under both basal and nitrogen-limited media. Enzyme abundance patterns illustrate carbon source–driven regulation of lipid-associated metabolic capacity, with nitrogen limitation modulating the magnitude of these responses. Color scales represent row-wise z-score–scaled group means. Created in BioRender. Oliveira Monteiro, L. M. (2026) https://BioRender.com/xdcnd0s.

## Fig. S12 ****Top 100 regulatory proteins identified exclusively on post-translational modification (PTM) datasets under different carbon sources.****

**a.** Venn diagram showing the overlap between regulatory proteins (RP) detected in global proteomics (n = 17), PTM-enriched dataset (n = 16), and exclusively detected in PTM-enriched dataset (44). **b.** Heatmap of PTM signals for the Top 100 post-translational modification (PTM) of regulatory proteins across glucose, glycerol, and xylose conditions, displaying relative modification levels across carbon sources. Color scales represent row-wise z-score–scaled group means.

## Fig. S13 ****Regulatory proteins identified across global proteomics and post-translational modification (PTM) datasets under different carbon sources**** and nitrogen availability****.****

**a.** Venn diagram showing the overlap between regulatory proteins (RP) detected in global proteomics (n = 17) and those identified in the PTM-enriched dataset (n = 16). Heatmap of RP abundance profiles derived from global proteomics across glucose, glycerol, and xylose growth conditions under basal and nitrogen-limited (NLIM) media. **b.** Heatmap of PTM signals for the overlapping RP, displaying relative modification levels across carbon sources, with annotations indicating PTM type (phosphorylation or cysteine oxidation). Highlighted RP represent regulators discussed in the subsequent section that are predicted to interact with enzymes from central carbon metabolism, including the TCA cycle, glycolysis, and lipid metabolic pathways. Color scales represent row-wise z-score–scaled group means. *Abbreviations: ACA1 = ATF/CREB activator 1, AATF = Apoptosis antagonizing transcription factor/protein transport protein, ARGR1 = Arginine metabolism regulation protein, CAF17/IBA57 = Iron-sulfur cluster assembly factor, CASP = Protein CASP, DOP1 = Protein DOP1, GPN3 = GPN-loop GTPase 3, HSF1/SKN7 = Heat shock transcription factor, MAK21 = Ribosome biogenesis protein, MBF1 = Multiprotein-bridging factor 1, MXR2 = Peptide methionine sulfoxide reductase, NAC = Nascent polypeptide-associated complex, NACB2 = Nascent polypeptide-associated complex subunit beta-2, NOT4 = General negative regulator of transcription subunit 4, RPA12 = DNA-directed RNA polymerase I subunit, RSC8 = Chromatin structure-remodeling complex protein, RTG3 = Retrograde regulation protein 3.*
